# Association between red blood cell distribution width and in-hospital mortality in acute myocardial infarction

**DOI:** 10.1097/MD.0000000000025404

**Published:** 2021-04-16

**Authors:** Sulan Huang, Quan Zhou, Ning Guo, Zhixiang Zhang, Li Luo, Yanlan Luo, Zuoan Qin, Liangqing Ge

**Affiliations:** Department of Cardiovascular, The First People's Hospital of Changde City, Changde, Hunan, China.

**Keywords:** acute myocardial infarction (AMI), in-hospital mortality, intensive care unit (ICU), red cell distribution width (RDW), repeated-measures analysis

## Abstract

Previous studies have shown an independent association between increased red cell distribution width (RDW) and mortality after acute myocardial infarction (AMI). However, evidence regarding the predictive significance of repeated measures of RDW in patients with AMI remains scarce. We aimed to investigate the association between the dynamic profile of RDW and in-hospital mortality in patients with AMI.

This was a cross-sectional study. We extracted clinical data from the Medical Information Mart for Intensive Care IIIV1.4 database. Demographic data, vital signs, laboratory test data, and comorbidities were collected from the database. The clinical endpoint was in-hospital mortality. Cox proportional hazards models were used to evaluate the prognostic values of basic RDW, and the Kaplan–Meier method was used to plot survival curves. Subgroup analyses were performed to measure mortality across various subgroups. The repeated-measures data were compared using a generalized additive mixed model.

In total, 3101eligible patients were included. In multivariate analysis, adjusted for age, sex, and ethnicity, RDW was a significant risk predictor of in-hospital mortality. Furthermore, after adjusting for more confounding factors, RDW remained a significant predictor of in-hospital mortality (tertile 3 vs tertile 1: hazard ratio 2.3; 95% confidence interval 1.39–4.01; *P* for trend <.05). The Kaplan–Meier curve for tertiles of RDW indicated that survival rates were highest when RDW was ≤13.2% and lowest when RDW was ≥14.2% after adjustment for age, sex, and ethnicity. During the intensive care unit stay, the RDW of nonsurvivors progressively increased until death occurred.

Our findings showed that a higher RDW was associated with an increased risk of in-hospital mortality in patients with AMI.

## 1 Introduction

Red blood cell distribution width (RDW) is a numerical measure of the variability in the size of circulating erythrocytes.^[1]^ Therefore, higher RDW values reflect greater heterogeneity in red blood cell size (anisocytosis), which is usually caused by perturbations in erythrocyte maturation or degradation.^[2]^ RDW has been reported as a coefficient of variation (percentage) of red blood cell volume.

High RDW values are representative of the biological effects of numerous endogenous and exogenous factors (i.e., age, sex, genetic background, inflammation, hormones, drugs, diet, exercise, hematological analyzers, and ranges of values) that modulate the biology and physiology of erythrocytes.^[3]^ Among the mentioned novel prognostic markers, RDW is routinely reported in complete blood count tests and serves as the coefficient for changes in mean corpuscular volume. Acute myocardial infarction (AMI) is a significant cause of morbidity and mortality in patients with coronary heart disease.^[4]^ Relationships between RDW and AMI, chronic heart failure, myocardial injury, peripheral artery disease, atrial fibrillation, hypertension, and stroke have been reported in numerous studies.^[3,5–11]^ Higher anisocytosis also significantly and independently predicted adverse outcomes in patients with these diseases. Furthermore, population-based studies have indicated that RDW is a predictor of all-cause mortality^[12]^ and cardiovascular mortality.^[13]^ Furthermore, it has been linked with mortality in patients with coronary artery disease,^[14]^ heart failure,^[15]^ ischemic stroke,^[16]^ acute kidney injury,^[17]^ cancer,^[18]^ and pulmonary hypertension.^[19]^ However, its current interest as a prognostic indicator of mortality and morbidity for cardiovascular diseases (CVDs) can be mitigated by fixing some critical aspects of RDW.

To the best of our knowledge, no previously published study has analyzed the relationship between repeated measures of RDW and in-hospital mortality among patients with AMI. In this study, we used the Medical Information Mart for Intensive Care III (MIMIC-III) database^[20]^ to examine the association between the dynamic profile of RDW and risk of in-hospital mortality in patients with AMI.

## 2 Methods

### 2.1 Data source

We extracted data from the MIMIC-III database, which included more than 40,000 intensive care unit (ICU) patients treated in a variety of critical care units (medical, surgical, coronary care, and neonatal) at the Beth Israel Deaconess Medical Center (Boston, MA) from 2001 to 2012.^[20,21]^ Our Access to the database was approved by the Institutional Review Boards of the Massachusetts Institute of Technology and Beth Israel Deaconess Medical Center after we completed the National Institute of Health's web-based course and passed the examination on Protecting Human Research Participants. Similar to the previous work,^[22–25]^ we extracted clinical data, including patient demographics, vital signs, laboratory test results, and other related parameters. To protect patient privacy, information regarding the included patients was hidden. One of the authors obtained access to this information and was responsible for data extraction (certification number: 6182750). The requirement for written informed consent was waived because of the retrospective nature of the study.

### 2.2 Population selection criteria

We restricted the search to adult patients (age≥18 years) with AMI using the International Classification of Diseases, Ninth Revision code. In total, 3209 adult AMI admissions were identified. Patients without RDW measurement during ICU admission were excluded from the study. The study population comprised of 3101 patients with AMI.

### 2.3 Data extraction

The Postgre SQL tool (version 9.6) was used to extract data from the MIMIC-III database. The extracted data comprised clinical parameters, laboratory parameters, demographic parameters, and scoring systems. The following comorbidities were included: congestive heart failure, pulmonary circulation disease, peripheral vascular disease, cardiac arrhythmias, other neurological diseases, hypertension, renal failure, liver disease, and diabetes. Laboratory measurements included RDW, serum creatinine, blood urea nitrogen (BUN), glucose, white blood cells (WBC), hematocrit, hemoglobin, platelets, serum sodium, serum potassium, troponin T, and creatine kinase isoenzyme. Sequential organ failure assessment (SOFA)^[26]^ and Simplified Acute Physiology Score II^[27]^ were obtained at the time of ICU admission. Baseline data were extracted within 24  hours after ICU admission. Dynamic data were collected during ICU admission. The clinical endpoint was in-hospital mortality among critically ill patients with AMI.

### 2.4 Statistical analyses

The baseline characteristics of all patients were stratified according to the RDW tertiles. Categorical variables were described as frequencies and percentages, and continuous variables were described using mean, median, and interquartile range values. We used the chi-square test for categorical variables and the Kruskal–Wallis test for continuous variables to compare groups. We used Cox regression to determine whether RDW was independently associated with in-hospital mortality among critically ill patients with AMI, and the results are presented as hazard ratios (HRs) with 95% confidence intervals (CIs). The lower-level group was considered the reference group. In Model I, covariates were adjusted only for age, sex, and ethnicity. In model II, covariates were adjusted for age, sex, ethnicity, length of stay in the ICU, serum potassium, troponin T, WBC count, systolic blood pressure (SBP), diastolic blood pressure, heart rate, respiratory rate, oxygen saturation (SPO_2_), congestive heart failure, cardiac arrhythmias, peripheral vascular disease, other neurological diseases, liver disease, renal failure, SOFA, and Simplified Acute Physiology Score II. We generated receiver operating characteristic (ROC) curves to measure the sensitivity and specificity of RDW and calculated the area under the curve (AUC) to ascertain the quality of RDW as a predictor of in-hospital mortality in patients with AMI. Moreover, we determined the relationship between RDW and classic scoring systems (SOFA and SAPSII scores). The Kaplan–Meier method (log-rank test) was used to plot the survival curves. Finally, we used a generalized additive model to compare trends in RDW over time among survivors and nonsurvivors, with an adjustment for potential confounders. We computed the delta RDW value as follows: absolute delta RDW= the mean RDW value of the first week - the mean RDW value of the second week.^[28]^

We conducted stratification analyses to investigate whether the effect of RDW differed across various subgroups, including age, sex, congestive heart failure, pulmonary circulation disease, peripheral vascular disease, cardiac arrhythmias, other neurological diseases, diabetes, hypertension, renal failure, and liver disease.

All data were analyzed using R software (version 3.42) and Empower Stats version 2.17.8 (http://www.empowerstats.com/cn/). Two-tailed probability values<5% were considered statistically significant, and all reported *P* values were 2-sided.

## 3 Results

### 3.1 Baseline characteristics of the study patients

**Figure 1:**
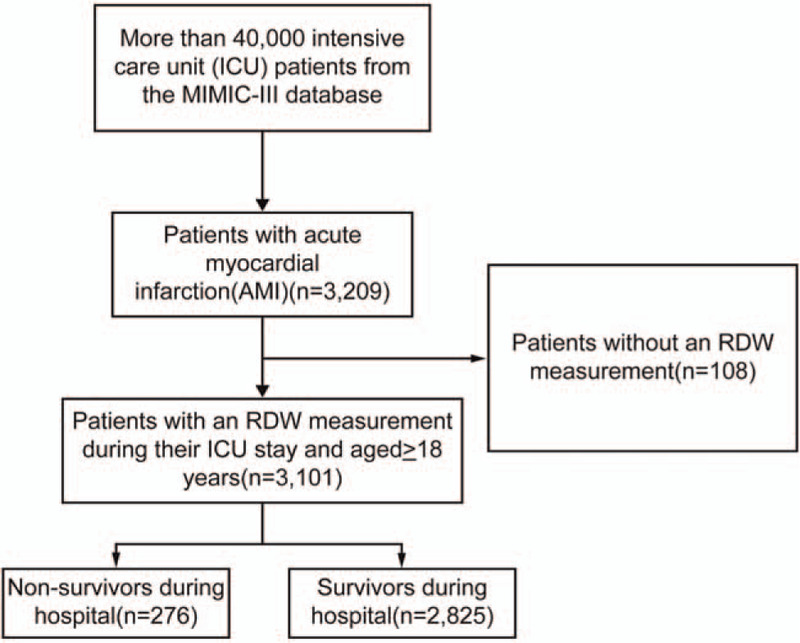
Flow chart of the patient selection process.

A total of 3101 eligible subjects were enrolled in the study, and 108 patients without RDW measurements were excluded from the study. In-hospital mortality for all patients with AMI was 8.9% (2825 survivors and 276 non-survivors) (Fig. 1). The baseline characteristics of the patients stratified by RDW tertiles are shown in Table 1. A total of 995 patients were in the low-RDW group (≤13.2), 996 patients were in the mid-RDW group (13.3%–14.1%), and 1110 patients were in the high-RDW group (≥14.2%). There were 1985 men and 1116 women, with a mean age of 69.4 ± 13.5 years. Of the patients enrolled, 2036 (65.7%) were White. Patients with high RDW values (RDW≥14.2%) were more likely to report a history of congestive heart failure, peripheral vascular disease, cardiac arrhythmias, diabetes, hypertension, liver disease, and renal failure. These patients were also more likely to receive percutaneous transluminal coronary angioplasty and coronary artery bypass grafting than those in the low- or mid-RDW groups. Patients in the high-RDW group were also older, had a higher divorce rate, and had a higher SBP, respiratory rate, serum potassium, BUN, serum creatinine, SOFA, and SAPSII, longer ICU length of stay, and higher in-hospital mortality. High-RDW patients also had a lower temperature, SPO_2_, WBC, hematocrit, hemoglobin, and troponin T levels compared with those in the low- or mid-RDW groups.

**Table 1:** Characteristics of the study patients according to tertile of RDW.

Variables	Tertile of RDW (%)			*P* value
	≤13.2 (n = 995)	≥13.3,≤14.1 (n = 996)	≥14.2 (n = 1110)	
Demographic				
Marital status, n (%)				<.001
Divorce	57 (5.73%)	45 (4.52%)	67 (6.04%)	
Married	604 (60.70%)	555 (55.72%)	584 (52.61%)	
Single	139 (13.97%)	145 (14.56%)	165 (14.86%)	
Widow	107 (10.75%)	184 (18.47%)	238 (21.44%)	
Other	88 (8.84%)	67 (6.73%)	56 (5.05%)	
Age (yrs), n (%)				<.001
<65	498 (50.05%)	391 (39.26%)	277 (24.95%)	
≥65	497 (49.95%)	605 (60.74%)	833 (75.05%)	
Gender, n (%)				<.001
Female	307 (30.85%)	345 (34.64%)	464 (41.80%)	
Male	688 (69.15%)	651 (65.36%)	646 (58.20%)	
Ethnicity, n (%)				<.001
White	615 (61.81%)	660 (66.27%)	761 (68.56%)	
Black	22 (2.21%)	31 (3.11%)	62 (5.59%)	
Other	358 (35.98%)	305 (30.62%)	287 (25.86%)	
Vital signs				
Heart rate, beats/min	96.0 (85.0,108.0)	96.0 (86.0,109.0)	96.0 (85.0,111.0)	.264
SBP, mm Hg	141.0 (127.0,154.0)	143.0 (130.0,158.0)	143.0 (130.0,158.0)	.001
DBP, mm Hg	80.0 (72.0,88.0)	81.0 (72.0,91.0)	79.00 (69.0,91.0)	.086
Respiratory rate, beats/min	25.0 (22.0,29.0)	26.0 (23.0,29.0)	26.0 (23.0,30.0)	<.001
Temperature, °C	37.4 (37.0,37.8)	37.4 (37.0,37.8)	37.2 (36.9,37.8)	<.001
SPO_2_, %	94.0 (92.09,6.0)	93.0 (91.0,95.0)	93.0 (90.0,95.0)	.006
Laboratory parameters				
WBC, 10^9^/l	10.7 (8.4,13.65)	10.8 (8.3,13.7)	10.3 (7.8,13.6)	.024
Hematocrit, %	39.0 (35.9,42.0)	38.3 (34.77,42.0)	34.7 (31.9,38.0)	<.001
Hemoglobin, g/dL	12.8 (11.7,13.9)	12.4 (11.0,13.8)	10.9 (9.8,12.2)	<.001
RDW, %	12.9 (12.6,13.1)	13.7 (13.5,13.9)	15.1 (14.5,16.2)	<.001
Platelet, 10^9^/L	194.0 (155.0,239.0)	195.0 (156.0,242.0)	191.0 (148.0,252.75.0)	.801
Serum sodium, mmol/L	136.0 (134.0,138.0)	136.0 (134.0,138.0)	136.0 (134.0,139.0)	.067
Potassium, mmol/L	3.7 (3.5,3.9)	3.7 (3.5,4.0)	3.8 (3.5,4.1)	<.001
BUN, mg/mL	17.00 (13.00,23.00)	20.00 (15.00,28.00)	28.00 (19.00,46.00)	<.001
Creatinine, mEq/L	0.9 (0.8,1.2)	1.00 (0.83,1.40)	1.30 (1.00,2.12)	<.001
CK-MB, ng/mL	9.7 (7.0,12.6)	9.7 (6.8,12.7)	9.4 (6.5,12.9)	.670
Glucose, mg/mL	110.0 (97.0,133.0)	112.0 (96.0,136.0)	110.0 (92.0,135.0)	.905
Troponin T, ng/mL	1.14 (0.34,3.45)	1.19 (0.33,3.78)	0.95 (0.30,2.71)	.016
Comorbidities				
Congestive heart failure, n (%)				<.001
No	668 (67.14%)	577 (57.93%)	408 (36.76%)	
Yes	327 (32.86%)	419 (42.07%)	702 (63.24%)	
Peripheral vascular disease, n (%)				<.001
No	929 (93.37%)	902 (90.56%)	928 (83.60%)	
Yes	66 (6.63%)	94 (9.44%)	182 (16.40%)	
Cardiac arrhythmias, n (%)				<.001
No	584 (58.69%)	545 (54.72%)	551 (49.64%)	
Yes	411 (41.31%)	451 (45.28%)	559 (50.36%)	
Other neurological diseases, n (%)				.152
No	952 (95.68%)	944 (94.78%)	1041 (93.78%)	
Yes	43 (4.32%)	52 (5.22%)	69 (6.22%)	
Diabetes, n (%)				<.001
No	728 (73.17%)	671 (67.37%)	647 (58.29%)	
Yes	267 (26.83%)	325 (32.63%)	463 (41.71%)	
Hypertension, n (%)				<.001
No	422 (42.41%)	348 (34.94%)	358 (32.25%)	
Yes	573 (57.59%)	648 (65.06%)	752 (67.75%)	
Liver disease, n (%)				<.001
No	978 (98.29%)	974 (97.79%)	1048 (94.41%)	
Yes	17 (1.71%)	22 (2.21%)	62 (5.59%)	
Renal failure, n (%)				<.001
No	948 (95.28%)	880 (88.35%)	796 (71.71%)	
Yes	47 (4.72%)	116 (11.65%)	314 (28.29%)	
Surgery				
PTCA, n (%)				<.001
No	796 (80.00%)	690 (69.28%)	780 (70.27%)	
Yes	199 (20.00%)	306 (30.72%)	330 (29.73%)	
CABG, n (%)				<.001
No	667 (67.04%)	643 (64.56%)	825 (74.32%)	
Yes	328 (32.96%)	353 (35.44%)	285 (25.68%)	
SOFA	2.0 (1.0,4.0)	3.0 (1.0,5.0)	4.0 (2.0,7.0)	<.001
SAPSII	29.0 (23.0,38.0)	31.5 (25.0,40.0)	37.0 (30.0,47.0)	<.001
LOS ICU, days	2.01 (1.18,3.42)	2.13 (1.26,4.01)	2.60 (1.45,4.89)	<.001
In-hospital mortality, n (%)				<.001
No	953 (95.78%)	919 (92.27%)	953 (85.86%)	
Yes	42 (4.22%)	77 (7.73%)	157 (14.14%)	

### 3.2 Association between RDW and AMI in-hospital mortality

**Figure 2:**
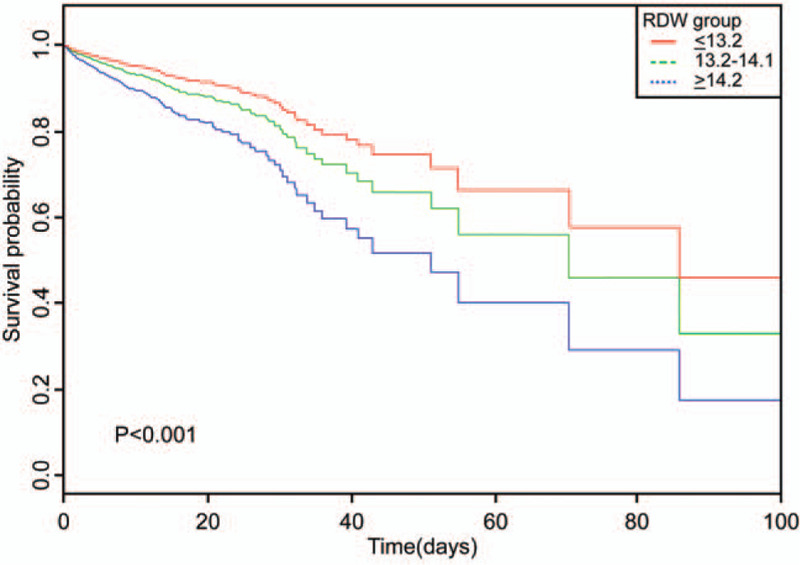
Kaplan–Meier survival curve for tertiles of red cell distribution width.

The Kaplan–Meier curve for the tertile of the RDW is shown in Figure 2. The figure indicates that survival rates were highest when the RDW was≤13.2% and lowest when the RDW was ≥14.2% after adjustment for age, sex, and ethnicity. RDW was able to distinguish between different survival statuses.

### 3.3 RDW as a predictor of in-hospital mortality

In model I, after adjustment for age, sex, and ethnicity, a higher RDW was associated with an increased risk of in-hospital mortality than for those in the first tertile (≤13.2) or quartile (≤13.0). In model II, after adjusting for more confounding factors, RDW was also an independent predictor of in-hospital mortality in critically ill patients with AMI (tertile 3 vs tertile 1: adjusted HR, 2.36; 95% CI, 1.39–4.01; *P* for trend<.05). A similar trend was observed in the RDW group inclusion according to quartiles (Table 2).

**Table 2:** HRs (95% CIs) for in-hospital mortality across groups of RDW.

	Nonadjusted		Adjust I		Adjust II	
RDW,%	HR (95% CIs)	*P* value	HR (95% CIs)	*P* value	HR (95% CIs)	*P* value
RDW	1.17 (1.10, 1.24)	<.0001	1.15 (1.08, 1.22)	<.0001	1.05 (0.97, 1.14)	.2326
Tertiles of RDW						
≤13.2	1.0		1.0		1.0	
≥13.3,≤14.1	1.55 (1.06, 2.26)	.0233	1.46 (1.00, 2.13)	.0505	1.44 (0.82, 2.54)	.2026
≥14.2	2.55 (1.81, 3.59)	<.0001	2.30 (1.62, 3.25)	<.0001	2.36 (1.39, 4.01)	.0016
*P* for trend	<.0001		<.0001		.004	
Quartiles of RDW						
≤13.0	1.0		1.0		1.0	
≥13.1,≤13.6	1.29 (0.81, 2.05)	.2890	1.26 (0.79, 2.01)	.3373	1.85 (0.86, 3.97)	.1155
≥13.7,≤14.5	1.90 (1.25, 2.89)	.0028	1.76 (1.15, 2.68)	.0092	2.83 (1.42, 5.66)	.0032
≥14.6	2.78 (1.86, 4.15)	<.0001	2.49 (1.66, 3.74)	<.0001	3.34 (1.19, 6.65)	.0006
*P* for trend	<.0001		<.0001		<.0001	

### 3.4 Subgroup analysis

Subgroup analysis of the association between RDW and in-hospital mortality was performed (Table 3). There were no interactions in most strata (*P* = .0974–.9634). Patients aged ≥65 years had a significantly higher risk of in-hospital mortality with aRDW≥14.2% (hazard ratio [HR] = 10.22; 95% confidence interval [95% CI, 4.17, 25.01; *P* = .0014]. Similarly, patients with liver disease showed an increased risk with a RDW≥14.2% (HR = 4.14, 95% CI, 1.34–12.82, *P* = .0312).

**Table 3:** Subgroup analysis of the associations between RDW and in-hospital mortality.

		Tertile of RDW (%)			
	n	≤13.2	≥13.3, ≤14.1	≥14.2	*P* for interaction
Age, yrs					.0014
<65	1166	1.0 (ref)	2.46 (0.85, 7.07)	7.93 (3.08, 20.45)	
>=65	1935	1.0 (ref)	7.61 (3.06, 18.92)	10.22 (4.17, 25.01)	
Gender					.9522
Female	1116	1.0 (ref)	2.55 (0.74, 8.80)	2.57 (0.78, 8.52)	
Male	1985	1.0 (ref)	2.26 (0.68, 7.54)	2.06 (0.66, 6.38)	
Ethnicity					.8250
White	2036	1.0 (ref)	1.61 (0.95, 2.71)	2.83 (1.76, 4.55)	
Non-White	1065	1.0 (ref)	2.73 (1.58, 4.71)	4.17 (2.54, 6.86)	
Congestive heart failure					.9221
No	1653	1.0 (ref)	3.50 (1.02, 12.05)	3.02 (0.92, 9.88)	
Yes	1448	1.0 (ref)	1.72 (0.52, 5.67)	1.72 (0.56, 5.32)	
COPD					.9634
No	2494	1.0 (ref)	2.78 (1.08, 7.14)	2.74 (1.15, 6.56)	
Yes	607	1.0 (ref)	3.06 (1.03, 9.07)	2.64 (0.92, 7.54)	
Peripheral vascular disease					.5100
No	2759	1.0 (ref)	2.62 (1.11, 6.20)	2.54 (1.09, 5.92)	
Yes	342	1.0 (ref)	2.58 (0.58, 11.40)	2.02 (0.75, 5.40)	
Cardiac arrhythmias					.0974
No	1680	1.0 (ref)	11.91 (1.45, 97.48)	12.44 (1.56, 99.12)	
Yes	1421	1.0 (ref)	12.72 (1.52, 106.54)	11.29 (1.43, 89.24)	
Other neurological diseases					.5978
No	2937	1.0 (ref)	2.90 (1.10, 7.66)	2.48 (0.99, 6.20)	
Yes	164	1.0 (ref)	9.11 (2.72, 30.51)	13.78 (4.50, 42.19)	
Hypertension					.4983
No	2857	1.0 (ref)	5.99 (1.17, 30.69)	5.18 (1.07, 25.12)	
Yes	244	1.0 (ref)	5.43 (1.13, 26.05)	5.50 (1.21, 25.12)	
Renal failure					.1901
No	2624	1.0 (ref)	2.51 (0.99, 6.34)	3.30 (1.35, 8.04)	
Yes	477	1.0 (ref)	4.63 (1.48, 14.48)	2.10 (0.79, 5.57)	
Liver disease					.0312
No	3000	1.0 (ref)	3.00 (1.29, 6.99)	2.15 (0.92, 4.99)	
Yes	101	1.0 (ref)	0.72 (0.11, 4.92)	4.14 (1.34, 12.82)	
Diabetes					.2020
No	2046	1.0 (ref)	1.62 (0.58, 4.51)	1.91 (0.77, 4.76)	
Yes	1055	1.0 (ref)	3.93 (1.46, 10.62)	2.85 (1.07, 7.63)	
PTCA					.5334
No	2266	1.0 (ref)	2.71 (0.99, 7.40)	3.17 (1.26, 8.00)	
Yes	835	1.0 (ref)	4.40 (1.44, 13.45)	2.83 (1.00, 7.99)	
CABG					.1169
No	2135	1.0 (ref)	2.24 (0.94, 5.35)	2.06 (0.89, 4.76)	
Yes	966	1.0 (ref)	1.08 (0.33, 3.51)	1.38 (0.43, 4.45)	

### 3.5 Prediction of in-hospital mortality

**Figure 3:**
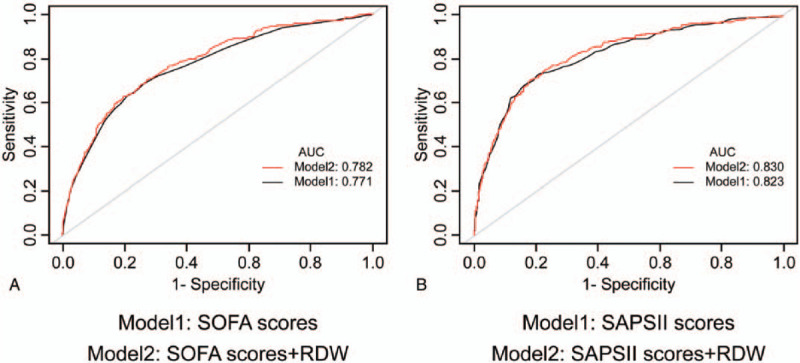
ROC curves for the prediction of in-hospital mortality in critically ill patients with AMI. A, The ability of SOFA scores and RDW plus SOFA scores to predict in-hospital mortality. B, The ability of SAPSII scores and RDW plus SAPSII scores to predict in-hospital mortality. AMI = acute myocardial infarction, RDW = red cell distribution width, ROC curve = receiver operating characteristic curve, SAPSII = Simplified Acute Physiology ScoreII, SOFA = sequential organ failure assessment.

ROC curves generated using the indicated variables (RDW, RDW plus SOFA scores, and RDW plus SAPSII scores) are plotted in Figure 3. The AUCs for SOFA scores were 0.771 compared with 0.782 for RDW plus SOFA scores (*P* < .001). Furthermore, the AUCs for SAPSII scores and RDW plus SAPSII scores were 0.823 and 0.830, respectively (*P* < .001).

### 3.6 Repeated-measure analysis

**Figure 4:**
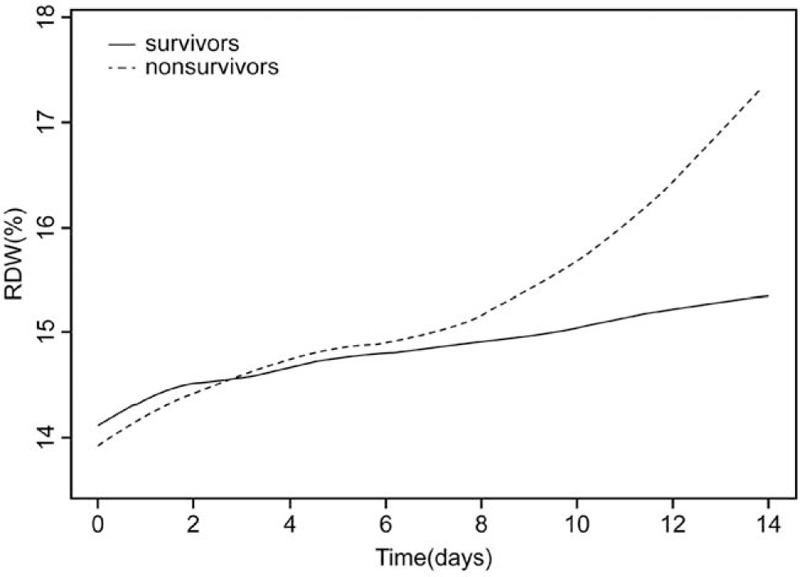
Dynamic profile of RDW in patients with AMI. Timeline charts illustrate RDW in patients with AMI (2825 survivors and 276 nonsurvivors) in ICU. The solid line shows the RDW level of survivors, and the dotted line shows the RDW level of nonsurvivors. *P* < .05 for nonsurvivors vs survivors. AMI = acute myocardial infarction, ICU = intensive care unit, RDW = red cell distribution width.

To determine the clinical features of AMI progression, repeated measures of RDW were tracked during ICU admission (Fig. 4). We observed changes in RDW over time between the 2 groups. During the ICU stay, the RDW of most patients increased over time. The RDW levels of the surviving group changed gradually and to a lesser extent than those of the nonsurvivors. However, the RDW of the nonsurviving group progressively increased in the ICU. In addition, we observed that the changes in RDW at 2 weeks after admission to the ICU were significantly different between the 2 groups. The delta RDW value was an independent predictor of in-hospital mortality in critically ill patients with AMI after adjusting for confounding factors and the HR was 1.57 (95% CI, 1.02–2.42, *P* = .0407). The delta RDW value of the nonsurviving group was higher than that of the surviving group (Supplementary Fig. 1).

## 4 Discussion

Our study found that RDW was an independent predictor of in-hospital mortality among patients with AMI. In addition, after adjusting for age, sex, ethnicity, and other confounding factors, a higher RDW remained a significant predictor of in-hospital mortality. Moreover, there were no significant interactions between RDW and most of the risk factors, and the stratified analysis of interactions indicated that a high RDW remained a predictor of in-hospital mortality. The AUC of the RDW plus SOFA scores or SAPSII scores had predictive value.

Previous studies supported a significant association between increased RDW values and CVDs and their prognosis,^[3]^ the correlation of which is even stronger than traditional factors.^[11]^ Tonelli et al ^[5]^ first investigated the association between elevated RDW and clinical outcomes in patients with coronary disease. They performed a post hoc observational analysis of 4111 participants, randomly divided into pravastatin and placebo groups, and used a Cox proportional hazards model to investigate the association between RDW levels and their outcomes. They revealed a graded independent relationship between high RDW levels and an increased risk of all-cause mortality in patients with coronary disease. Dabbah et al^[29]^ conducted a study that followed 1709 patients with AMI for a mean duration of 27 months and found that there was a graded, independent association between increased RDW and mortality after AMI. Khaki et al^[30]^ followed 649 patients with AMI for 6 months and found that the 6-month mortality rate was significantly higher in patients with high RDW than in those with a low RDW. Similar to these results, our study also showed a positive correlation between RDW and in-hospital mortality of AMI. Survival rates were highest when the RDW was≤13.2% and lowest when the RDW was ≥14.2% after adjusting for age, sex, and ethnicity. As a result, our study presents a crucial novel finding that supplements those of previous studies.

RDW is a novel predictive marker and an independent risk factor that plays a significant role in assessing the severity and progression of cardiovascular diseases. However, the mechanisms underlying the association between RDW and the prognosis of CVDs remain unclear. Factors impairing bone marrow hematopoietic function play an integral role in this process. These factors are identical to the factors that worsen the prognosis of patients with coronary artery disease. Other existing hypotheses mainly focus on microvascular disorders,^[31]^ anemia,^[32]^ inflammatory cytokines,^[33]^ oxidative stress,^[34]^ free cholesterol,^[35]^ thrombosis,^[36]^ nutritional deficiency,^[11]^ and neurohumoral and adrenergic systems.^[37]^ This may help explain the potential link between RDW and in-hospital mortality among patients with AMI.

Several possible mechanisms have been proposed in previous studies. Increasing RDW during the hospital course may reflect the bone marrow response to the cumulative influence of multiple humoral mediators in the setting of AMI. The production of red blood cells by bone marrow is regulated by the hormone erythropoietin. Plasma erythropoietin levels increased in the early phase of AMI, independent of hemoglobin levels.^[38]^ Based on these studies, we suggest a combined evaluation of RDW with other emerging biomarkers related to the prognosis of CVDs, including telomere length of leukocytes, circulating nucleated red blood cells, and endothelial progenitor cells. In contrast, the level of cholesterol from the erythrocyte membrane has been confirmed to be positively associated with RDW in patients with coronary disease.^[35]^ Zhong et al ^[39]^ also verified that the level of total cholesterol correlated with the severity of coronary artery disease. Presumably, the effect of RDW on myocardial infarction might be supported by the cholesterol pathway.

The dynamic profile of RDW findings was tracked in patients with AMI upon ICU admission. In the survivors, the levels of RDW were slightly elevated; however, for the nonsurvivors, the levels of RDW were higher and continued to increase until death occurred. This further indicates that RDW is closely related to in-hospital mortality in patients with AMI. In the authors’ opinion, multiple measurements of RDW during hospitalization are essential. The predictive value of the dynamic profile of the RDW was greater than that of single measurements. Future studies on RDW may allow its use as a biomarker profile. An appropriate algorithm could be created, which could help in the diagnosis and predict the prognosis of different CVDs. Furthermore, the physio-pathological scenario of CVDs is extremely complex; therefore, the singular monitoring of red blood cells cannot represent the most accurate strategy for predicting cardiac disorders. As a result, RDW cannot potentially be used as a prognostic indicator alone, but would likely need to be combined with other cardiac biomarkers, such as natriuretic peptides, atrial natriuretic peptide, and B-type natriuretic peptide.^[3]^

Our study has several strengths. First, to the best of our knowledge, this is the first study to analyze the association between the dynamic profile of RDW and in-hospital mortality in patients with AMI using repeated-measure analysis. Additionally, the large number of subjects with AMI is an important strength of the present study.

The results of the present study must be interpreted in the context of their limitations. First, this was a cross-sectional study from a single center, limiting inferences about the causality of the results. Second, elevated RDW levels are associated with certain conditions such as reticulocyte count, erythropoietin levels, iron, vitamin B12, folate, and hemolysis. These parameters were not measured in this study. Finally, the follow-up length of the mortality varied. We analyzed in-hospital mortality. Therefore, large prospective multicenter studies and follow-ups are needed to confirm these results in the future.

In conclusion, our findings showed that a higher RDW was associated with a risk of in-hospital mortality in critically ill patients with AMI. We believe that RDW is a cheap and readily available predictive marker in contrast to other novel markers of in-hospital mortality.

## Author contributions

**Conceptualization**: Sulan Huang, Quan Zhou, Ning Guo, Liangqing Ge.

**Data curation:** Quan Zhou, Zuoan Qin.

**Formal analysis:** Sulan Huang, Quan Zhou, and Ning Guo.

**Funding acquisition:** Ning Guo, Zhixiang Zhang, Liangqing Ge.

**Investigation:** Sulan Huang, Liangqing Ge, Li Luo, Yanlan Luo.

**Methodology:** Sulan Huang, Quan Zhou.

**RResources:** Sulan Huang, Quan Zhou.

**Supervision:** Liangqing Ge.

**Writing – original draft:** Sulan Huang, Quan Zhou.

**Writing – review & editing:** Ning Guo, Liangqing Ge.

## Supplementary Material

Supplemental Digital Content
