# Supplementary material for: Association between red blood cell distribution width and in-hospital mortality in acute myocardial infarction
Source: Medicine (Baltimore). 2021 Apr 16;100(15):e25404. doi: 10.1097/MD.0000000000025404 (PMC8052072; doi:10.1097/MD.0000000000025404)

**Supplemental material**

In Sup Fig. 1, we can observe different delta RDW value between the two groups. The value of non-surviving group[0.243(0.080,0.533)]was higher than the surviving group[0.100(0.000,0.267), *P*<.001].


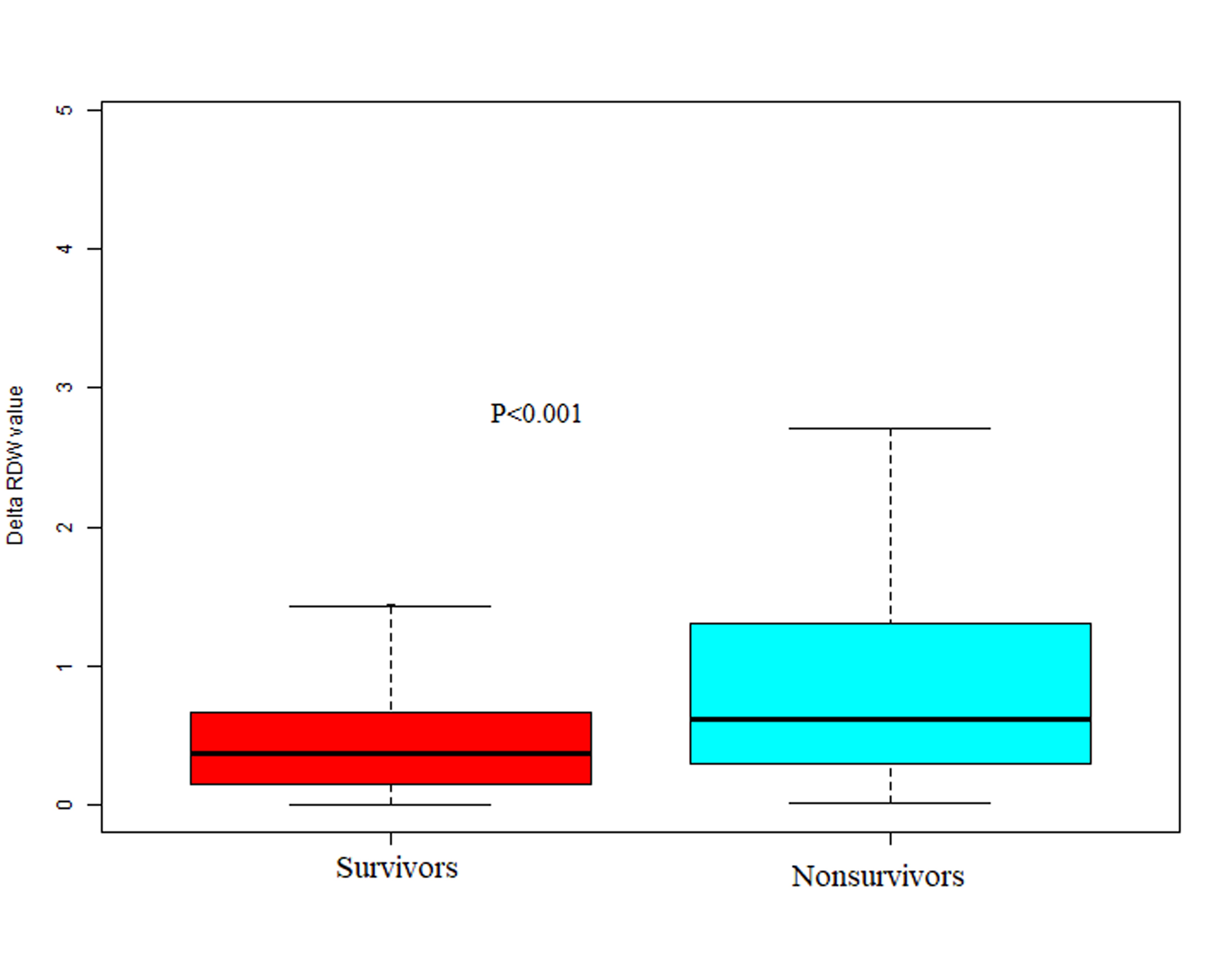

Supplement: Supplemental Digital Content [file medi-100-e25404-s001.doc]
